# Issues in healthcare services in Malaysia as experienced by Japanese retirees

**DOI:** 10.1186/s12913-016-1417-3

**Published:** 2016-05-05

**Authors:** Ayako Kohno, Ghazali Musa, Nik Daliana Nik Farid, Norlaili Abdul Aziz, Takeo Nakayama, Maznah Dahlui

**Affiliations:** Department of Social and Preventive Medicine, Faculty of Medicine, University of Malaya, Kuala Lumpur, Malaysia; Department of Health Informatics, Kyoto University School of Public Health, Kyoto, Japan; Faculty of Business and Accountancy, University of Malaya, Kuala Lumpur, Malaysia; Julius Centre, University of Malaya (JCUM), Faculty of Medicine, University of Malaya, Kuala Lumpur, Malaysia

**Keywords:** Japanese retirees, Healthcare, Qualitative study, Focus group discussion, Malaysia my second home

## Abstract

**Background:**

Worldwide, international retirement migration is growing in its popularity and Japanese retirees choose Malaysia as their most preferred destination. This study examines the pertinent issues related to healthcare services as experienced by Japanese retirees in this country.

**Methods and results:**

From January to March 2015, we conducted focus group discussions with 30 Japanese retirees who live in Kuala Lumpur and Ipoh. Guided by the social-ecological model, we discovered seven pertinent themes: ‘language barriers’,’healthcare decisions’, ‘medical check-ups’,‘healthcare insurance’, ‘nursing and palliative care’, ‘trust and distrust of healthcare services’, and ‘word-of-mouth information’.

**Discussion:**

We identified seven pertinent issues related to healthcare services among Japanese retirees in Malaysia, of which four are especially important. These issues are explained as integrated themes within the social-ecological model. Language barriers prohibit them from having difficulty accessing to healthcare in Malaysia, but lack of will to improve their language skills exist among them. For that reason, they rely heavily on word-of-mouth information when seeking for healthcare. As a consequence, some develop feelings of trust and distrust of healthcare services. In addition, we have identified the needs for provide nursing and palliative care among Japanese retirees in Malaysia.

**Conclusion:**

Based on the magnitude of the discussion, we concluded that there are four crucial healthcare issues among Japanese retirees; ‘language barriers’, ‘trust and distrust of healthcare services’, ‘word-of-mouth information’ and ‘nursing and palliative care’. We propose that further dialogue by healthcare stakeholders should be carried out to improve further the healthcare service provisions for Japanese retirees in Malaysia.

## Background

Japanese retirees who live in foreign countries have increased in recent years. In Malaysia, according to the statistics from the Malaysia My Second Home (MM2H) Centre, a total of 3,872 MM2H visas has been issued to Japanese nationals, during the period of 2002 when the program started, until November 2015 [1]. This figure only represents the number of persons given permission to stay in Malaysia with an MM2H visa. The characteristics of the visa is that its holders can be accompanied by their families, such as spouses, parents and unmarried children under twenty-one years old [2]. Therefore, the number of Japanese retirees in Malaysia is likely to be much bigger than the number of visas issued.

This form of international retirement migration (IRM) is growing in popularity worldwide and will most probably continue to remain so as a popular option for retirement in the future, especially for those who seek a different lifestyle during retirement [3]. Among Japanese, IRM is widely known as *Long-stay* [4]. According to the annual survey conducted by the Long-Stay Foundation in Japan, Malaysia has been the number one destination country in the ranking for long-stays among Japanese for eight consecutive years since 2006 [5]. The successful applicants of an MM2H visa can obtain social visit passes with multiple entries to Malaysia for up to ten years [1]. The permitted stay period is much longer compared with other neighboring Southeast Asian countries such as Indonesia, Thailand, and the Philippines [4].

Although the number of Japanese retirees who live in Malaysia is increasing, there is hardly any research carried out to understand the healthcare issues experienced by them. Research on the motivation and attractions to retire in Malaysia has been conducted from a tourism perspective which includes some brief descriptions of their healthcare concerns, but none of the literature focuses on healthcare as a core research objective [6, 7]. Therefore, the overall research objective of this study is to examine the pertinent issues related to healthcare services as experienced by Japanese retirees in Malaysia.

### Background information

Retirement migration is a movement of people from their home country to live abroad for an extended stay, a process or an event that is influenced by several factors such as personal resources and their characteristics, community and housing characteristics, and social factors and support networks [8–10]. Retirees in Europe and North America migrate for various reasons, such as seeking a warmer climate, a new lifestyle, cheaper living costs and cultural experiences [11–13]. International Retirement Migration (IRM) also frequently occurs in the form of “return migration”, for people who immigrated to Europe and North America in the early stages of their lives return to their respective native countries for retirement. Another form of migration is the movement to live in a country a short distance away from theirs, such as Germans immigrating to Austria, or Americans immigrating to Canada. The geographical and cultural proximities clearly play some significant roles in choosing the destination for IRM [13, 14].

Warnes [13] categorised retirees who prefer to live in the countries with warmer climates into healthy migrants and migrants with chronic illnesses. Healthy migrants are those who perform long distance migration, but are healthy both mentally and physically. On the other hand, migrants with chronic illnesses are those who cannot bare the harshness of cold weather, and this drives them to migrate to warmer climate countries. For Japanese retirees who live in Malaysia, both of these categories are prevalent [6]. There are only a few studies carried out on the topic related to the social and cultural issues of Japanese retirees living abroad [2, 4, 15]. Ono conducted a qualitative study on Japanese retirees in Malaysia [4]. The study provided an historical overview of Japanese long-stay tourism, and revealed the main motivations and the lifestyles of Japanese retirees living in the country. The main motivations were for economic and social security reasons and seeking a better quality life. Miyazaki conducted a study on Japanese retirees’ migration to Asia and Oceania [15]. The study placed emphasis on the need for elderly care services. Trained nurses and care-givers are crucial to support the lives of elderly Japanese retirees who remain in the destination countries. Stapa et al. investigated the degree to which Japanese retirees are willing to learn Malay language or English for communicating with local people [2]. Results showed that English was the language of choice (and not Malay), when Japanese retirees communicate with doctors in the hospital to explain their health ailments. Most Japanese retirees live in urban areas, where English is widely spoken among residents either for daily life or in business communication. The study also reported a rather rigid integration process of Japanese retirees into the Malaysian society. They claimed to experience difficulty in their efforts to integrate; and some, surprisingly, refused to integrate by intentionally keeping distance from the local community. The word ‘contemplation’ was used to describe the slow process of Japanese retirees getting used to the new environment and culture in Malaysia [2]. Living among Japanese circle and community, they maintain their value as being Japanese in a foreign country. The attitude of Japanese retirees varies slightly depending on where they live. Those who live in the capital city favour convenience and excitement that the urban life can offer, whereas those who live outside Kuala Lumpur prefer a more relaxed lifestyle in a quiet environment. Some of them live in a resort complex with golf courses, allowing them to indulge in golfing daily.

Fukahori et al. carried out a survey on the healthcare services provided for Japanese retirees in Thailand, from the perspective of both patients and healthcare providers [16]. The language barrier was identified as an obstacle when Japanese retirees visited the hospitals. They recommended the need for improvement in communication between Japanese retirees and healthcare providers, by providing opportunities for healthcare providers to take communication and cultural educational training. Fukahori et al. also raised the issue of the need for terminal care including palliative care for Japanese retirees in Thailand.

Wong and Musa conducted a qualitative study on the motivations of international retirees living in Malaysia [6]. They found the seeking of self-fulfillment needs was the dominant motivation for the international retirees in the country. For those who had favourable experience in the past by visiting the country and had fun experience, that experience leads to their motivation to regain satisfaction by choosing Malaysia for place of retirement. Previous satisfactory travel experiences in Malaysia normally preceded the decision to choose the country as a retirement destination. Healthcare issues were raised by some participants, claiming arthritis as the reason to move to a warmer climate. Participants also praised the high quality medical services which are available in Malaysian hospitals.

Back in Japan, company workers and their families aged 40 and above are encouraged by their employers to take an annual medical check-up, and this has become their habit. An open personality of the Japanese elderly, such as intellectual curiosity and a preference for various experiences is reported to be associated with the participation in mass health check-ups in Japan [17]. While living overseas, they look for inexpensive and thorough medical check-ups, if possible equivalent to what is available in Japan. With respect to the health insurance system in Japan, generally, for those who are members of the Japan’s National Health Insurance (NHI) system, and those who had paid for their insurance premium, they are only required to pay thirty percent of the actual hospital cost at the hospitals in Japan [18]. This system is also applicable for Japanese retirees who live overseas, including in Malaysia.

With the exception of Fukahori et al. [16], research related to the healthcare of Japanese retirees overseas is scarce to date. In Malaysia, despite its popularity as a retirement migration destination among the Japanese, there have been no study which examined the pertinent issues related to healthcare services in the country as experienced by Japanese retirees.

## Methods

### Setting

We conducted a total of six focus group discussions (FGDs) with Japanese retirees living in Malaysia. Kuala Lumpur and Ipoh were purposely sampled for the study, in view of large Japanese communities who live at the locations. Five focus group discussions were conducted in Kuala Lumpur, of which four were at the Japan Club and one at the participants’ condominium. Another focus group discussion was conducted at the Ipoh Japan Club. The Japan Clubs have facilities which are safe and comfortable, and are a common venue for the retirees’ socialisation.

### Participants

A total of thirty Japanese retirees participated in focus group discussions. Participants were recruited by convenience sampling, mainly during a seminar held at the Japan Club of Kuala Lumpur about retirement life in Malaysia. The primary author (AK) personally invited the Japanese retirees who attended the seminar to participate in the research. Through these participants AK also identified and invited their friends via snowball sampling to take part in the discussion. In addition, participants were also recruited through a member-only social network site ((http://malaysia-navi.vivian.jp/sns/)) on which an invitation for a focus group discussion was announced on this website. This social network site is for Japanese retirees living in Malaysia and Japanese who currently live in Japan and wish to immigrate to Malaysia for their retirement. The inclusion criterion was that the participants must be Japanese retirees who have lived in Malaysia for retirement purposes at least six months at the time of participating in the discussion.

### Procedure

Focus Group Discussion (FGD) was chosen as the data collection method, and we used a semi-structured interview to answer and elaborate on each question. Focus group discussion allows the dynamic exchange of ideas among participants, reflecting on what others say and further expressing and sharing their opinions about healthcare experiences in Malaysia. A classroom setting was provided with tables and chairs, where participants, a facilitator and a note taker sat facing each other. This environment was created to allow participants to feel the proximity to each other during the focus group discussions in a relaxed environment, and at the same time have a certain degree of formality while involved in the dialogue. Each discussion session lasted for an average of two hours, and was conducted in the Japanese language. The meeting started out with a friendly introduction of each participant to the other group members. The participants were then asked to share their feelings about healthcare in Malaysia, including their worries and concerns. A prepared set of questions was asked by the facilitator, and subsequent probing questions were also directed to the participants when it was recognised by the facilitator as appropriate timing within the flow of the conversation. With participants’ permission and after explaining the purpose of the study, the focus group discussions were audio-recorded. The transcriptions were made verbatim in the Japanese language first, and then later translated to English. The translation was done by the primary author (AK). The transcribed texts, the memos and reflective journals were the three types of data used for analysis. Codings were made to a transcript from the first focus group discussion, and then the same set of codes was reviewed against the rest of the transcripts for each discussion. Additional new codes were created, when the subsequent transcripts touched upon a subject which had not been discussed during previous discussions. After all the codings were created, a list of codes was reviewed against all the transcripts to check for validity.

### Research tools

We prepared a discussion guide prior to conducting the focus group discussion to explain to the participants the aim of the study, the procedure of the focus group discussion as well as the protection of personal information attained during the discussion. The questions were directed to the participants during each focus group discussion by the facilitator. Some of the questions were improvised and canvassed as the conversation continued. In addition, some participants also asked other participants questions, to express their curiosity of what others thought about healthcare issues they had experienced in the country. This research is inductive and exploratory in nature, to allow for variety of views to be expressed by the participants. The prepared set of questions were pilot-tested on two Japanese retirees living in Kuala Lumpur by conducting in-depth interviews, and small modifications to the wording was made, based on the feedback received from these interviews. Table 1 shows the list of the prepared set of questions used for the FGDs.

**Table 1:** Questions asked during FGD with Japanese retirees in Malaysia

What are the worries and concerns you have in relation to your healthcare services in Malaysia?
What do you consider to be the most attractive part of medical services in Malaysia?
Is the word-of-mouth from your friends who are Japanese retirees in Malaysia helpful in deciding about your healthcare services in Malaysia?
What kind of information do you need to help you in making decisions about which hospitals to go to?
Do you feel a sense of social solidarity with your current life in Malaysia? If so, with what (or with who) do you feel the solidarity?
How do you consider your sense of connectedness with the people in Malaysia?
What do you do to maintain your health while living in Malaysia? Is there a special type of food you consume or exercise you do?
How long do you plan to live in Malaysia?
Which hospital have you used, or intend to use; private or public? What are the reasons for your choice?

A simple questionnaire survey was also carried out on the participants at the beginning of the focus group discussion. This was to collect the participants’ demographic information which is summarised in Table 2.

**Table 2:** Demographic information of Japanese retirees participated in FGDs

Criteria	
Age (average) in years	54–79 (65.2)
Gender	
Male	14
Female	16
Final School Attended	
University	18
High School	8
Special School	3
Junior High School	1
Years living in Malaysia (average)	0.5–20 (5.5)

### Conceptual framework

**Fig. 1:**
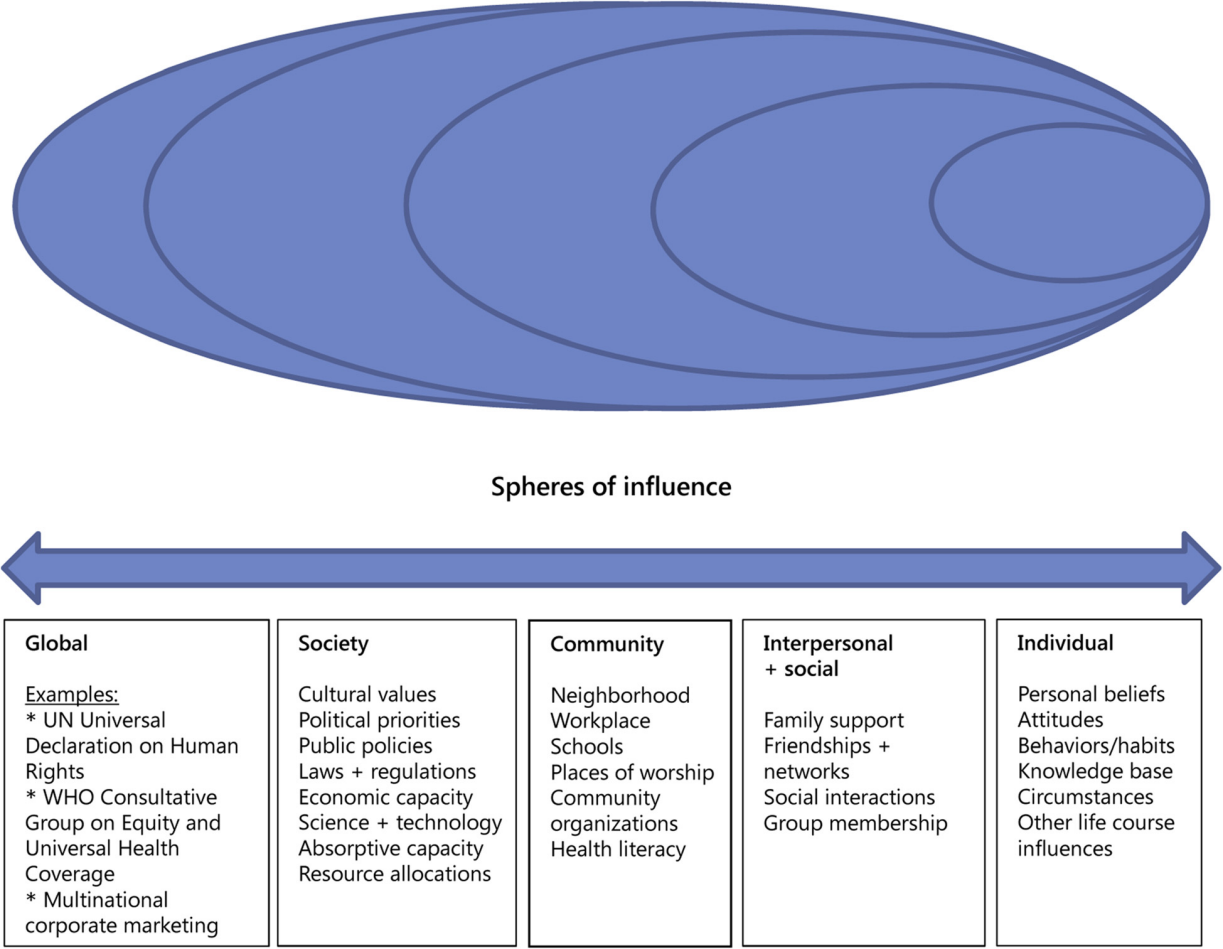
Social-ecological model – spheres of influence. Adapted from “Primary Health Care and Public Health: Foundations of Universal Health Systems” by Franklin White, 2015, Medical Principles and Practice, 24:103–116. Copyright 2015 by S. Karger AG, Basel. Adapted with permission

In this study we chose the social-ecological model (refer Fig. 1) to illustrate the factors of human development such as cultural, sociological and environmental in multiple layers and in an interdependent manner [19]. White explained the social-ecological model in the context of primary healthcare and an integrated healthcare system [20]. The model was originally developed by Bronfenbrenner, and later many scholars and organisations utilised it in various fields such as violence prevention, cancer treatment, and others [21]. The social-ecological model was also used to examine the retirement expectations of Australian baby boomers [22].

The social-ecological model describes the interplay between multiple spheres of influence; individual, interpersonal and social, community, society and global. The individual layer describes personal beliefs, attitudes, behaviour and knowledge of the individuals. The interpersonal and social layers are concerned with the closest social circle of the individuals such as family, friends and social and group networks. The community layer governs the social relationship such as schools, workplace and neighborhoods. The society layer explains aspects such as cultural values, public policies, laws and regulations and resource allocations. Lastly, the global layer includes aspects including human rights, healthcare coverage and multinational corporate marketing. The social-ecological model displays the fact that human relationship is interdependent within the multiple layers of the system. Bronfenbrenner proposed the use of this model “to analyse systematically the nature of the existing accommodation between the person and the surrounding milieu.” [19]. In this study, we used the model to guide data analysis and to illustrate the interconnectedness of the dimensions within the sphere of healthcare services in Malaysia.

### Ethical considerations

The study was approved by the Medical Ethics Committee, University of Malaya Medical Centre (UMMC), Malaysia with MECID.NO: 201411–815. Prior to conducting the FGDs, the participants were fully informed about the study’s objectives. The study’s voluntary nature was clarified, and the right to refuse participation was guaranteed to the participants. We also asked the participants to sign their consent to participate in the discussion, and ensured the protection of their personal information. All participants of this study had agreed to sign the consent sheet and written consents were obtained from all participants.

### Data analysis

Guided by a Qualitative Analysis Guide of Leuven (QUAGOL) [23], the transcribed data was analysed using the thematic analysis method [24, 25]. Primary author (AK) created the first initial coding of all focus group discussions, which were later reviewed, commented on and modified by three other authors (GM, ND and NAA). Following multiple discussions, the researchers came to an agreement of the final codings. We used QSR NVivo 10 software to organise the data and create the codes and themes. During the coding process, the question was asked regarding what the participants had talked about, and emphasis was given to identifying the underlying meanings of the statements. Codes were created with the consideration of perspectives held by participants related to healthcare issues, healthcare processes, activities, relationships and the social and medical structures. We created 127 codes during the coding process. Then, the layering of codes were conducted to aggregate similar codes and reduce redundancy. Thirty seven of such sub-category codes were created and analysed further to reduce them into themes. We identified seven themes which were most frequently mentioned as the main findings of this research. Quotations from the transcribed data were carefully selected that best illustrated the themes.

## Results

### Demographic information

The participants consisted of 14 males and 16 females whose ages were between 54 to 79 years old. The average age for the participants was 65.2 years old and the average duration of stay was 5.5 years. The majority of the participants (18 out of 30) had a tertiary education. Table 2 shows the demographic information of the participants.

### Themes of healthcare services issues

There are seven crucial themes which emerged from the analysis. These are ‘language barriers’, ‘healthcare decisions’, ‘medical check-ups’, ‘healthcare insurance’, ‘nursing and palliative care’, ‘trust and distrust of healthcare services’, and ‘word-of-mouth information’. We present the results according to the dimensions of the social-ecological model which consists the layering of individual, interpersonal and social, community, society, and global.

## The individual layer

### Healthcare decisions

Decisions about healthcare among Japanese retirees in Malaysia fit within the individual layer of the social-ecological model. Some Japanese retirees, who were either required to or have a possibility to undergo surgery in Malaysia, often considered going back to Japan to have the procedure. They feel more secure about going through a big event in their home country.

*So, well, if I have a major disease, I have already decided to receive treatment in Japan. I feel safer about having the treatment in my own country.* (FGD3, female)

However, in risky situations, some chose to have treatment in Malaysia:

*Our doctor suggested that my husband should have a pacemaker. We said that we’d like to consider whether to have it here or in Japan. But if something happened in the plane on the way back to Japan? We consulted our local friends, and finally decided to have it here* (FGD6, female).

### Trust and distrust of healthcare services

We observed that some Japanese retirees have issues with ‘trust and distrust of healthcare services’ in Malaysia, which also fit within the individual layer of the social-ecological model. Retirees were not only concerned about the quality of medical staff such as doctors and nurses, but also other equipment operators and cleaning staff in the hospitals. Many aspects of these concerns were also interconnected with the results from their interactions with the hospital staff, which were often marred with communication difficulties. Language barriers prevents them from having satisfactory communication.

*They may have excellent medical equipment, but I am worried about the quality of medical staff. Can they operate the machine effectively? Can they interpret the diagnostic results the same way as doctors in Japan? What are the perceptions and practices of hygiene among nurses?* (FGD3, female)

On the other hand, some Japanese retirees trust the doctors and medical services provided in Malaysia. The reasons are the acceptance of their health condition, previous satisfactory experience and interaction with medical staff, and prior familiarity and knowledge with the medical services in Malaysia.

*Without medical knowledge I am sure I would misunderstand some details which doctors tried to tell me, even with the help of interpreters. But I am quite relaxed about it. I trust the skills of the doctors here which are wonderful. And, the medical equipment, MRI and others, they are wonderful. I worked in a pharmaceutical company before, so (compared to Japan), the hospitals here are not inferior.* (FGD6, male)

## The interpersonal and social layers

### Language barriers

For ‘interpersonal and social’ layers, two important themes or issues related to healthcare services have been discovered. These are ‘language barriers’ and ‘word-of-mouth information’. Participants mentioned their inability to communicate in English when visiting hospitals in Malaysia. Although English is the main language of communication in most private hospitals in Kuala Lumpur and Ipoh, Japanese retirees prefer to visit the establishments which have either a Japanese doctor or provide Japanese interpreters. Often they are unable to inform medical staff of their health symptoms in English. They prefer their complaints to be heard and understood by doctors who speak the native Japanese language. Without it they fear misdiagnosis and wrong treatment decisions by the local doctors.

*I think it is better to speak in your mother tongue. If I say I have pain, then they say I must undergo surgery, then I am in trouble. If I say itchy pain, then well, that is not the type for which surgery is needed, it could be treated. So, if the judgment of the doctor depends on what I say, I feel safer to consult a Japanese doctor.* (FGD4, male)

Ironically, some Japanese retirees are reluctant to improve their ability to speak English while living in Malaysia. Even though they have learned the English language during secondary school, their mastery is insufficient to hold daily conversation. Reluctant to assimilate with other communities, Japanese retirees who live in big cities as Kuala Lumpur and Ipoh, spend most of their day among other Japanese retirees, watch news in Japanese through Japanese cable TV, and read Japanese newspapers and websites. Therefore, there is no incentive for them to adapt to their new environment where thinking and communicating in English is required.

### Word-of-mouth information

Word-of-mouth information with regard to healthcare is the most trusted source among Japanese population, which is crucial for healthcare service decisions. They prefer and trust any information about hospitals, treatments and other healthcare services from other Japanese retirees who live in the country, rather than the local people. Japanese retirees largely share the same feelings and cultural values of what is considered as common sense and the quality of medical services offered in the hospitals in Malaysia. Their justification for choosing to trust Japanese and not local people is based on their inability to interact proactively with local people to gain the type of information required. Among Japanese retirees, it is perceived that, in order to demonstrate high quality medical services, doctors and nurses should be tender, take their time to listen to patients’ worries and concerns, and be polite.

*We feel Japanese people have a better grasp of our needs with regards to medical services in Malaysia. Thus we prefer to ask Japanese retirees who live in Malaysia rather than local people.* (FGD1, female)

*If they (the Japanese nearby) say the hospital is good then we go to the hospital; and if they say it is not good, we will not go.* (FGD3, female)

## The community layer

### Nursing and palliative care

A compelling issue for some of Japanese retirees was nursing and palliative care. For Japanese retirees in Malaysia, they perceive palliative care as the treatment for those who are terminally ill. Whereas, nursing care is a service for someone with relatively mild chronic symptoms and difficulty in performing daily activities due to old age, and they need assistance to carry out daily activities. Even though at the moment, they are healthy enough and able to take care of themselves, in the next five to ten years, they eventually become weaker, and some worry about becoming demented, during which they would need full time nursing care. Some retirees are planning to stay in Malaysia indefinitely, and do not intend to return to Japan. In order to spend the end stage of their lives with dignity and comfort, they may need to live in nursing homes, where high quality services are provided. In doing so, Japanese retirees prefer and have high expectations to be in institutions where high quality services are available in terms of detailed attentions and care to each elderly person.

*I am contemplating to live for the rest of my life in Malaysia. It is good so long as we can do things by ourselves, but as there maybe times when we need to rely on someone more or less, to attend to our needs and live a meaningful life. Is there such a facility which allows us to live in such a way in Malaysia?* (FGD2, male).

It is common in Malaysia that elderly people are taken care of at home by their family members or with the support of caregivers. However, Japanese retirees in Malaysia are mainly living without their children, therefore, they will not have the support from young family members. In Japan, many are nuclear families, so people cannot expect to have the support of the family members when they are old. Therefore, most Japanese elderly wish to enter nursing homes. However, nursing homes in Malaysia are still scarce in their supply, especially the high quality nursing establishments as envisaged by Japanese retirees.

### The society layer

#### Medical check-ups

At the society layer, the decision to have medical check-ups again will be influenced by other Japanese retirees (society) who live in Malaysia. For the procedure, Japanese retirees were divided as to whether having it in Malaysia and to return to Japan for the procedure. The key factor in choosing to have a medical check-up in Malaysia is the quality of services provided in the hospitals.

*Up until now, I’ve been having medical check-ups once a year in Japan, and now I am looking for a medical institution in Malaysia which can offer me a similar level of service.* (FGD1, male)

Among Japanese people, there is a general acceptance of the necessity to have medical check-ups annually. Therefore there is a high demand among Japanese retirees to carry out medical check-ups at the hospitals or clinics in Malaysia. Again, at the society layer, Japanese retirees will rely on word-of-mouth information from Japanese retirees of the best possible venues to carry out medical check-ups.

*In order to detect health’s signs and symptoms, with quantitative figures, we need to have the medical check-up. So, for our household, we have the medical check-ups every year.* (FGD1, male)

### The global layer

#### Healthcare insurance

At the global layer, features of Japanese insurance coverage were discussed as an important advantage to the retirees. The system allows Japanese nationals who live overseas to get reimbursement of the medical cost which is paid overseas via National Health Insurance coverage. In addition, some other types of insurance were also utilised by Japanese retirees and some purchased the overseas trip insurance which has a wide coverage of injuries and sudden illnesses, but is not applicable to chronic illnesses, or any illnesses that one had prior to subscribing to the insurance [26]. Some other common types of insurance are attached to the credit card as collateral. Although the coverage period is limited to generally three to six months after departure from Japan, those card holders can take advantage of the overseas trip insurance services attached to the credit card. However, the issue is, some of the older Japanese retirees, who are more than 70 years old, have difficulty subscribing to any private insurance, as there is no such service provided to this age group. Therefore, some Japanese retirees live in Malaysia without any insurance coverage. When hospital treatment is required, they have to pay for all of the costs accruing at their own expense.

*Well, my husband is also above 70 (years old). We cannot have the overseas insurance. So, in the office where I am purchasing my insurance, they say they can do something about this, but my husband says “I don’t become sick, I do not die.” And the other day, he became sick, and it was terrible…* (FGD2, female)

The users of the health insurance need to know what requirements and limitations are there when using the health insurance that they are subscribed to. The differences in medical systems between Malaysia and Japan also need to be understood by Japanese retirees in Malaysia, in order to apply for a reimbursement.

## Discussion and conclusion

**Fig. 2:**
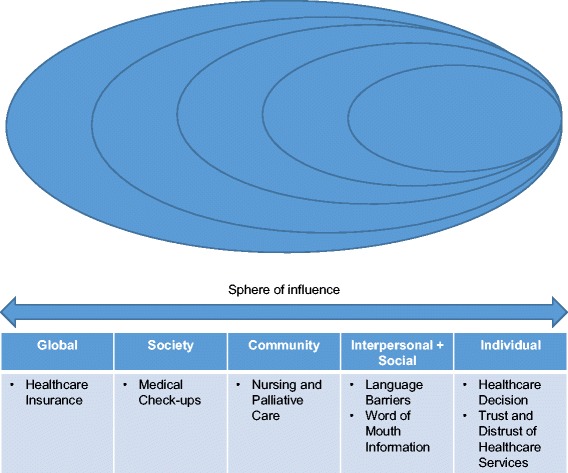
Social-ecological model with seven pertinent issues from this study

We identified seven pertinent issues (themes) related to healthcare services among Japanese retirees in Malaysia, which include negative and positive aspects (Refer Fig. 2).

The themes are integrated within the social-ecological model. Firstly, at an individual layer, ‘healthcare decisions’ among retirees favor treatment to be carried out in Japan, except for simple health symptoms and emergency situations. This decision, which is based on personal beliefs and behaviours of Japanese retirees, are influential factors, may derive from prior knowledge and upbringing, and is called *habitus* by Pierre Bourdieu’s conceptualisation [27, 28]. Simultaneously, Japanese retirees developed ‘trust and distrust of healthcare services’ from their own personal experience, and communication with other Japanese retirees. At the interpersonal and social layers, pertinent issues related to healthcare services are ‘language barriers’, and ‘word-of-mouth information’. Limited English proficiency and the lack of will to learn English result in ‘language barriers’. This provides compounding effects on any effort to integrate with the local community and culture. Therefore many Japanese retirees rely on ‘word-of-mouth information’ to facilitate their choices in healthcare services consumption. At the community layer, some Japanese retirees plan to live in Malaysia for good. However, many are skeptical in Malaysia’s readiness to provide ‘nursing and palliative care’ with similar standards to Japan. At the society layer, Japanese retirees are accustomed to performing personal and family ‘medical check-ups’ regularly. The challenge is to look for reliable hospitals and clinics, which have modern medical equipment and medical staff who could interpret diagnostic results similar to the Japanese doctors. At global layer, Japanese national ‘healthcare insurance’ also partly covers the medical expenses in Malaysia.

Among the seven themes from research analysis, the following four have more significant managerial implications and deserve further discussion. These are ‘language barriers’, ‘word-of-mouth information’, ‘trust and distrust of healthcare services’, and ‘nursing and palliative care’. Factors of ‘language barriers’ and ‘word-of-mouth information’ determine the perception of ‘trust and distrust of healthcare services’ and should be managed accordingly. Meanwhile, the need for ‘nursing and palliative cares’ is identified as a new product and services to be developed to cater for not only Japanese retirees but also other Malaysia My Second Home participants and Malaysian society at large. Of all the themes discovered in this study, perhaps the ‘language barrier’ is the most serious issue which affects healthcare utilisation and satisfaction among Japanese retirees living in Malaysia. Language barriers result in the reliance of ‘word-of-mouth information’ and recommendations when making healthcare decisions, and also the formation of ‘trust and distrust of healthcare services’.

The preference to live within the comfort of the Japanese social system and community deepens the seriousness of the ‘language barriers’. The retirees frequently congregate at Japan Clubs in Kuala Lumpur and Ipoh for social interaction, and this isolates them from other Malaysian communities and societies. This is more so among Japanese retirees who live in Kuala Lumpur, within the more individualistic culture and society. By contrast, the situation is slightly different for Japanese retirees living in Ipoh. Given the small size of the Japanese community and the higher proportion of Chinese-Malays living in the region, communalistic culture is more prevalent as compared with Kuala Lumpur. There seems to be some interaction and assistance from local residents to Japanese retirees when utilising healthcare services.

While living in Malaysia, Japanese retirees who cannot speak English fluently, think and gather information in the Japanese language. The use of Japanese media communication (e.g. Internet, radio and TV) and the preference for socialisation only among Japanese retirees in Malaysia, allow them to get by in daily life thinking and speaking only in their native language. When they visit hospitals or clinics, their health services consumption will be facilitated by Japanese interpreters who are more likely available in the higher-class private hospitals. In the medical establishments, even though sufficient level of conversation can be carried out between Japanese patients and physicians using the interpreters, retirees still perceived that they have missed out on a lot of information conveyed to them through this triadic communication. The common limitation of the triadic communication is that, it could easily create misunderstanding, and consequently breed distrust among Japanese retirees in Malaysian medical establishments.

Hudelson pointed out that, ideally, physicians should be well trained to understand communication with foreign patients to overcome potential cross-cultural communication barriers between them and foreign patients [29]. Hudelson also highlighted the role of medical interpreters as cultural mediators, who need to incorporate cultural aspects in the communication process [29]. Participating Japanese retirees stated their hesitation to visit hospitals or clinics for simple symptoms like having flu or body itchiness. Their worries are mainly about not being able to communicate effectively in English, the availability of Japanese interpreters and lack of information in the Japanese language, all of which lead them to doubt the trustworthiness of healthcare services in the country. They only visit medical establishments when the health situation becomes critical.

Stemming from the combination of ‘language barriers’ and ‘word-of-mouth information’, the healthcare seeking behaviour of Japanese retirees in Malaysia is biased in particular ways. The sense of distrust by Japanese retirees is not a strong sentiment of dislike or discontent, but rather a subtle feeling, a sense of not receiving appropriate treatment or not getting sufficient response from physicians. Hsieh stated that in interpreter-mediated medical encountering process, the meaning of words, especially medical terminologies, must be negotiated among the patient, physician and interpreter [30]. However, in many of the actual cases of medical consultations received by Japanese retirees in Malaysia, the language barriers limits this negotiation, resulting in a more likely engagement of a one way dialogue between local physicians and Japanese retiree patients. For this reason, in Malaysia, Japanese retirees prefer to rely on word-of-mouth information from other Japanese retirees when making healthcare decisions, and consult a Japanese doctor at the hospital. Some retirees however, trust the healthcare services in Malaysia. The trust stemmed from innate feelings and evaluative judgement that each individual makes, based on gathered information and prior experience. Berger stated that when word-of-mouth information is utilised in interpersonal communication, it is goal driven, meaning underlying motives exist to achieve that goal by speaking about a particular issue [31]. There are five categories of such motives suggested by Berger; impression management, emotion regulation, information acquisition, social bonding and persuasion. All of these motives fit well to the situation of Japanese retirees who rely heavily on word-of-mouth information about healthcare. By conversing among Japanese retirees about healthcare and medical issues, they can fulfill the needs of seeking advice, satisfying emotional distress, justifying their own decisions, as well as trying to persuade others. Word-of-mouth information compensates their lack in ability to communicate with physicians and nurses in English.

Stapa et al. typified the communication and cultural assimilation among Japanese retirees living in Malaysia as contemplation. It will take a long time for Japanese retirees to adapt to the new living environment and culture in Malaysia [2]. They are not willing to drastically change their lifestyle and behaviours after arriving in the country, so they make every effort they can to maintain their Japanese lifestyle, including their attitudes and behaviours when interacting with doctors and nurses in Malaysia. The behaviour of Japanese retirees in Malaysia is perceived as common from the viewpoint of the Japanese, but may appear rather strange or even unintelligible for the non-Japanese. The word “contemplation” best describe their behaviour of those who tend to delay the decision making in accepting new culture and healthcare services. Japanese share the sense of virtue to put on hold their decisions, looking for the safest and the most feasible option, especially when they do not have strong confidence in making a decision. They only make healthcare decisions upon receiving the most convincing and trustworthy information, and this is often gained from the Japanese communication network.

‘Nursing and palliative care’ is another important aspect that emerged in this study. Retirees expressed their interests in and concerns to know more about the available options for nursing and palliative care in Malaysia. As Fukahori et al. mentioned, there is a need for palliative care in the case of Japanese retirees in Thailand [16], and this need is also evident among Japanese retirees in Malaysia. Japanese retirees’ expectations for palliative care are not about how to cure the disease, but how to live their lives with dignity and comfort to the very end. As this service is intricately linked to culture and values of the people who receive care, effective communication is crucial. The mastery of the Japanese language is the key in providing the required services. Japanese retirees look for an excellent environment which is safe, comfortable and supportive in Malaysia. There is a high expectation for establishing nursing homes in Malaysia that can provide palliative care which has Japanese communication preference with the residents. The need for amenity services among elderly parents of retirees, was also mentioned in the study by Wong and Musa [8]. The provision of such services may need financial and decisional supports from both private and public sectors. The advanced palliative and nursing care will not only be attractive to Japanese retirees but also to other retirees in general.

The study is the first to examine the healthcare issues among Japanese retirees living in Malaysia. One of its limitations, however, is that we only interviewed the participants in two major cities in Malaysia - Kuala Lumpur and Ipoh. The former is Malaysia’s biggest city in Peninsular Malaysia, while the latter is the third biggest. The inclusion of Penang, which is the second biggest city and the second most populous destination patronised by Japanese retirees, could certainly provide a richer and more balanced knowledge of healthcare services issues among them. However, the focus group discussion could not be arranged in Penang due to the preference for personal privacy not to be disturbed among retirees. Additionally authors were constrained by temporal and financial limitations. Being qualitative in nature, there is a limitation to generalise the study’s findings. Focus group discussions which chose participants with convenience sampling only recruited among those who were within the social circle of Japanese retirees. Generally, during focus group discussions, opinion leaders often monopolise the conversation and influence the study findings. However, during all focus group discussions in this study, the main author (AK) did not experience this issue, perhaps owing to the communalistic culture of Japanese people who are rather reversed in giving their opinions and more willing to also listen to others during the discussions. Future research should continue in quantitative form or using the positivistic approach to quantify the magnitude of the issues and validate the qualitative findings. Another immediate study that should be carried out is the satisfaction of Japanese retirees towards received medical care in Malaysia.

In conclusion, this qualitative research describes the healthcare services issues among Japanese retirees using the social-ecological model. Language barriers, trust and distrust of healthcare services, word-of-mouth information and nursing and palliative care are important themes discovered in the analysis. It is recommended that further studies should investigate and validate these themes. Also, economic, political and diplomatic considerations should be instituted to establish Japanese style nursing homes in Malaysia, as well as the support from both private and public sectors, to secure comfortable and conducive long-stay living environment for and welcoming more Japanese retirees in Malaysia. Our findings suggest the need for further academic research and political dialogue between Malaysia and Japan to improve the healthcare provisions among Japanese retirees in Malaysia.

## Availability of data and materials

Extra data can be accessed via the Dryad data repository at http://datadryad.org/resource/doi:10.5061/dryad.kn37p.

## Abbreviations

FGDs: focus group discussions
IRM: international retirement migration
MM2H: Malaysia my second home
NHI: National Health Insurance
QUAGOL: Qualitative Analysis Guide of Leuven
UMMC: University of Malaya Medical Centre
